# Green Carbon Dots from Pinecones and Pine Bark for Amoxicillin and Tetracycline Detection: A Circular Economy Approach

**DOI:** 10.3390/jox15020043

**Published:** 2025-03-11

**Authors:** Saheed O. Sanni, Ajibola A. Bayode, Hendrik G. Brink, Nils H. Haneklaus, Lin Fu, Jianping Shang, Hua-Jun Shawn Fan

**Affiliations:** 1 College of Chemical Engineering, Sichuan University of Science & Engineering, Zigong 643000, China; mosqit.saheed@gmail.com (S.O.S.); bayodea@run.edu.ng (A.A.B.); susefulin@163.com (L.F.); welsons@126.com (J.S.); fan27713@yahoo.com (H.-J.S.F.); 2 Department of Chemical Engineering, Faculty of Engineering, Built Environment and Information Technology, University of Pretoria, Pretoria 0028, South Africa; 3 Department of Chemical Sciences, Redeemer’s University, P.M.B. 230, Ede 232101, Nigeria; 4 Td-Lab Sustainable Mineral Resources, Universität für Weiterbildung Krems, Dr. Karl-Dorrek-Straße 30, 3500 Krems an der Donau, Austria; 5 Unit for Energy and Technology Systems—Nuclear Engineering, North-West University, 11 Hoffman Street, Potchefstroom 2520, South Africa

**Keywords:** carbon dots, pine cone, pine bark, amoxicillin, tetracycline, fluorescent quenching

## Abstract

Over the years, the abuse of antibiotics has increased, leading to their presence in the environment. Therefore, a sustainable method for detecting these substances is crucial. Researchers have explored biomass-based carbon dots (CDs) to detect various contaminants, due to their low cost, environmental friendliness, and support of a circular economy. In our study, we reported the synthesis of CDs using pinecones (PCs) and pinebark (PB) through a sustainable microwave method. We characterized the PCCDs and PBCDs using X-ray diffraction, Raman spectroscopy, Transmission Electron Microscope, and Fourier transform infrared, Ultraviolet-visible, and photoluminescence (PL) spectroscopy. The PCCDs and PBCDs were tested for the detection of amoxicillin (AMX) and tetracycline (TC). The results indicated that the sizes of the PCCDs and PBCDs were 19.2 nm and 18.39 nm, respectively, and confirmed the presence of the 002 plane of the graphitic carbon structure. They exhibited excitation wavelength dependence, good stability, and quantum yields ranging from 6% to 11%. PCCDs and PBCDs demonstrated “turn-off” detection for TC and AMX. The limits of detection (LOD) for TC across a broader concentration range were found to be 0.062 µM for PCCDs and 0.2237 µM for PBCDs. For AMX detection, PBCDs presented an LOD of 0.49 µM.

## 1. Introduction

Antibiotics like amoxicillin and tetracycline are extensively used in human and veterinary medicine to combat bacterial infections [1,2]. However, their overuse and improper disposal lead to their accumulation in environmental matrices, such as soil and water bodies, resulting in antibiotic resistance, hepatotoxicity, nephrotoxicity, gastrointestinal effects, photosensitivity in humans, and ecological imbalance [3,4]. The World Health Organization (WHO) underscores the critical need to reduce the inappropriate use of antibiotics like amoxicillin (AMX) and tetracycline (TC), as they contribute to the growing threat of antimicrobial resistance (AMR) [5], hence calling for the need for the development of efficient, cost-effective, and environmentally friendly methods for detecting these antibiotics in environmental matrices.

Carbon-based nanomaterials have emerged as versatile tools in the field of analytical sensing due to their exceptional chemical, physical, and optical properties [6,7]. Among these, carbon dots (CDs) have garnered significant attention because of their high biocompatibility, tunable fluorescence, ease of functionalization, and sustainable synthesis methods [7,8]. Derived from a wide array of natural and waste materials, CDs have found applications in diverse fields, including bioimaging [9], drug delivery [10], catalysis [11], and environmental monitoring [12]. A particularly promising direction is their use in detecting pharmaceutical contaminants, such as antibiotics, which pose significant environmental and public health challenges [13].

Various methods have been employed for the detection of AMX and TC, including high-performance liquid chromatography (HPLC) [14], electrophoresis, colorimetric detection [15], titrimetric methods, mass spectrometry (MS) [16], enzyme-linked immunosorbent assays (ELISA) [17], spectrophotometric methods [18]**,** and electrochemical sensors [19]. These techniques offer high sensitivity and specificity, their major shortcomings are that they require expensive instrumentation, extensive sample preparation, a specific operating temperature, skilled personnel, and are difficult to operate [20,21]. Additionally, these methods are not always suitable for on-site or real-time monitoring, calling for a method that can aid rapid diagnostics.

Carbon dots (CDs) have emerged as a superior sensing material due to their remarkable photoluminescence properties, cost-effective synthesis, biocompatibility, and ability to be derived from renewable natural materials [22,23]. CDs exhibit excellent chemical stability, low cost, good reliability, excellent dispersibility in water-tunable fluorescence, and functional groups on their surface, which allow for selective interactions with AMX and TC [24,25]. These interactions often lead to fluorescence quenching or enhancement, forming the basis for simple, rapid, and sensitive detection. Furthermore, CDs synthesized from waste materials such as pinecones and bark contribute to sustainability, making them an eco-friendly and efficient alternative for AMX and TC sensing.

Over the years, researchers have explored natural sources (biomass or agro-waste) such as pinecones and pine bark, which offer an abundant and renewable precursor material for synthesizing CDs [26,27], in a bid to promote a circular economy. These biomass-derived sources are rich in lignin, cellulose, and other carbon-rich compounds, which can be transformed into fluorescent CDs through methods like hydrothermal or pyrolysis processes [28]. Utilizing such renewable precursors not only aligns with sustainable development goals, but also adds value to agricultural and forestry waste, mitigating environmental concerns.

Pinecone (PC) and pine bark (PB) are abundant in the environment, and they are used for decorations, paper production, fertilizers, and resin oils, etc. [29]. Pinecone and pine bark synthesized CDs (PCCDs and PBCDs) exhibit unique structural and photoluminescent properties, making them particularly suitable for sensing applications [30,31]. Their fluorescence can be tailored via doping or surface functionalization, enabling selective interactions with specific molecules such as amoxicillin and tetracycline. These interactions often result in fluorescence quenching or enhancement, forming the basis for a sensitive and selective detection mechanism. However, the application of PCCDs and PBCDs to detect TC and AMX has not been reported yet.

This study presents a sustainable and efficient approach for synthesizing CDs using a green microwave-assisted method with pinecone and pine bark as the carbon sources, eliminating the need for additional chemical agents. The structural and morphological properties of the resulting PCCDs and PBCDs were thoroughly characterized using various analytical techniques, while their optical properties were evaluated through spectrophotometry. The fluorescence quenching method demonstrated the high sensitivity and selectivity of these materials for detecting AMX and TC, highlighting their potential for real-world water quality monitoring. Notably, PCCDs and PBCDs showed strong performance in TC detection, with PBCDs exhibiting exceptional specificity for AMX, reinforcing their applicability in environmental sensing. This research contributes to sustainable water management and public health monitoring, aligning with SDG 3 (Good Health and Well-Being) and SDG 6 (Clean Water and Sanitation).

## 2. Materials and Methods

### 2.1. Sample Collection and Pretreatment

The waste biomass of pinecones and pine bark was collected from the Vaal University of Technology campus, Vanderbijlpark, South Africa. Analytical grade chemicals such as rhodamine B (99%), the nitrate salts of zinc (Zn^2+^), cadmium (Cd^2+^), iron (III) (Fe^3+^), iron (II) (Fe^2+^), nickel (Ni^2+^), and mercury (Hg^2+^) were obtained from Sigma-Aldrich. Pharmaceuticals such as tetracycline (TC), chloramphenicol (CLM), amoxicillin, (AMX), ciprofloxacin (CIP), sulfamethazole (SMZ), and ethanol were all obtained from Merck Chemicals. These chemicals mentioned above were utilized without further purification, and ultrapure water was used throughout the experiments.

### 2.2. Synthesis of PCCDs and PBCDs

**Figure 1:**
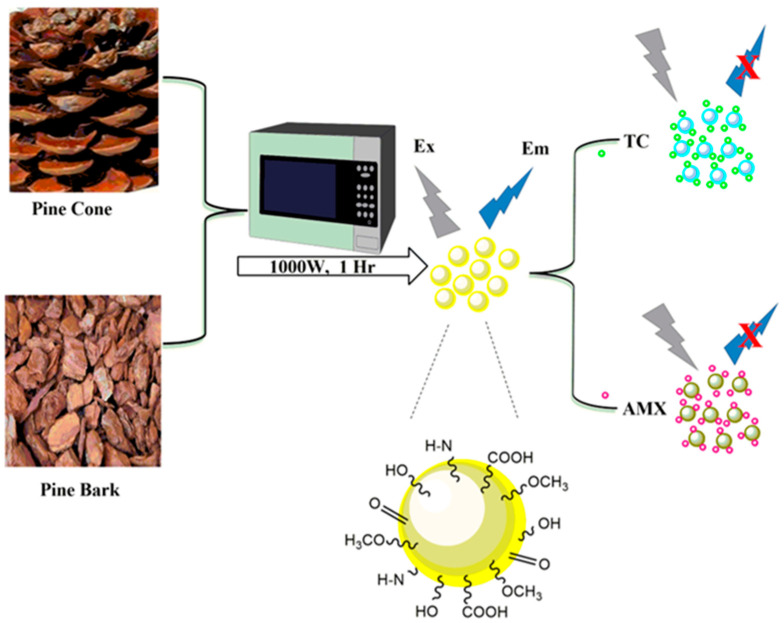
Schematic diagram illustrating the preparation process of PCCBs and PBCDs, and their sensing process.

The preparation procedure of PCCDs and PBCDs using the microwave-assisted method is shown in Figure 1. Firstly, the well-dried PC and PB biomasses were ground with a coffee grinder and sieved to obtain a fine powder. Then, 5 g of sieved PC/PB biomass underwent microwave pyrolysis for 1 h at 1000 W (periodic cooling at 10 min), under an inert atmosphere through nitrogen gas purging. The obtained carbonized materials (3 g each) were dispersed in 150 mL ultrapure water, sonicated at 45 min, and then centrifuged for 60 min at 10,000 rpm. Next, the solutions were filtered through a 0.22 μm filter membrane, whilst the solid PCCDs and PBCDs were obtained freeze-drying [32].

### 2.3. Instrumentation

Transmission Electron Microscope (TEM) and Scanning Electron Microscope (SEM) images of the CDs were recorded using the Tecnai 20 Transmission Electron Microscope (FEI Company, Hillsboro, OR, USA) and ZEISS Ultra/Plus FEG-SEM (JEOL Ltd., Tokyo, Japan). The powder X-ray diffraction patterns for the prepared CDs were obtained via a Bruker diffractometer AXS (Billerica, MA, USA), at a scan rate of 0.02° 2θ s^−1^ in the range of 5–60° at room temperature. Fourier transform infrared spectrometer (FTIR) values of the PCCDs and PBCDs were recorded using a Perkin Elmer spectrum 400 (Waltham, MA, USA) in the wavenumber range from 500 to 4000 cm^−1^. A Horiba LabRAM HR Evolution (Kyoto, Japan) measured the Raman spectra of the prepared CDs, and the scattering range was 600–2000 cm^−1^. The thermal analysis was carried out with the Perkin Elmer STA 6000 thermal simultaneous analyzer (Waltham, MA, USA), operating at a 10 °C/min heating rate under a nitrogen purge stream from 30 to 900 °C under 19.8 mL/min. The Ultraviolet-visible (UV-VIS) absorption spectra of the prepared CDs were measured using a Double beam UV-VIS spectrophotometer between 200 and 800 nm. The fluorescence spectra of the CDs were examined using a Jasco FP-8600 spectrophotometer in the range of 200 to 1000 nm (Midrand, South Africa).

### 2.4. Detection of TC and AMX

The fluorescence detection experiments were conducted using the nitrate salt of zinc (Zn^2+^), cadmium (Cd^2+^), iron (III) (Fe^3+^), iron (II) (Fe^2+^), nickel (Ni^2+^), mercury (Hg^2+^), tetracycline (TC), chloramphenicol (CLM), amoxicillin, (AMX), ciprofloxacin (CIP), and sulfamethazole (SMZ). The metal ion and antibiotic stock solutions (20 mM) were prepared in ultrapure water. Then, 1.0 mL of each metal cation and antibiotic mentioned above was mixed with 1.0 mL of PCCDs/PBCDs (1 mg/mL) and 3 mL of PBS buffer solution (pH 7) at room temperature for selectivity studies. The solution mixture was measured after interaction for 5 min, and its corresponding fluorescence emission intensities were obtained simultaneously. However, 1.0 mL of TC and AMX at different concentrations (5, 10, 20, 30, 40, 50, 75, and 100 μM) was utilized for sensitivity studies. The excitation wavelength was fixated at 360 nm and 345 nm for PCCDs and PBCDs, respectively. The limit of detection of PCCDs and PBCDs with the antibiotics (TC/AMX) was calculated using the Stern–Volmer relationship [33].

## 3. Results

### 3.1. Characterization Analysis

**Figure 2:**
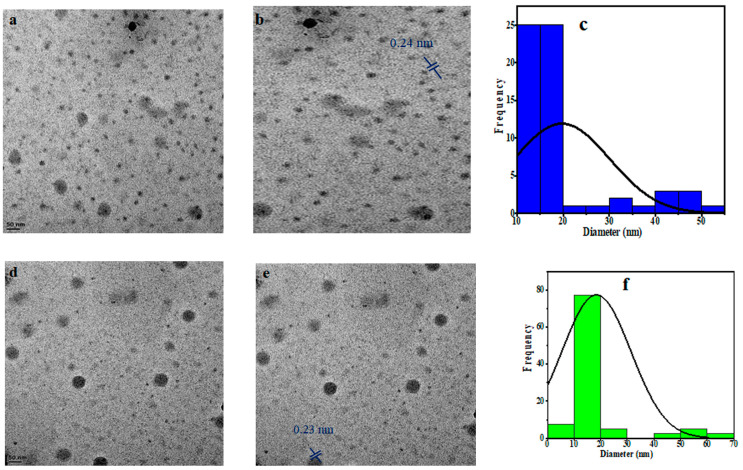
(**a**,**d**) TEM picture, (**b**,**e**) HRTEM picture, and (**c**,**f**) size distribution histogram of the PCCDs and PBCDs.

The PCCDs and PBCDs, synthesized from pine tree wastes through microwave pyrolysis without any chemical additives, are presented in Figure 1. The shape, size distribution, and morphology of PCCDs and PBCDs from the TEM analysis are depicted in Figure 2a–f. The PCCDs and PBCDs are spherical and there is no evidence of agglomeration (Figure 2a,d). The HRTEM for both CDs (Figure 2b,e) evidenced an average lattice spacing of 0.24 nm, ascribed to the (100)-plane characteristic of regular graphite [34]. The CDs obtained from pine trees have an average size range of between 3.00 and 59.90 nm, while the hydrodynamic diameters for PCCDs and PBCDs (Figure 2c,f) are 18.3 ± 0.8910 nm and 18.1117 ± 0.2783 nm, respectively.

**Figure 3:**
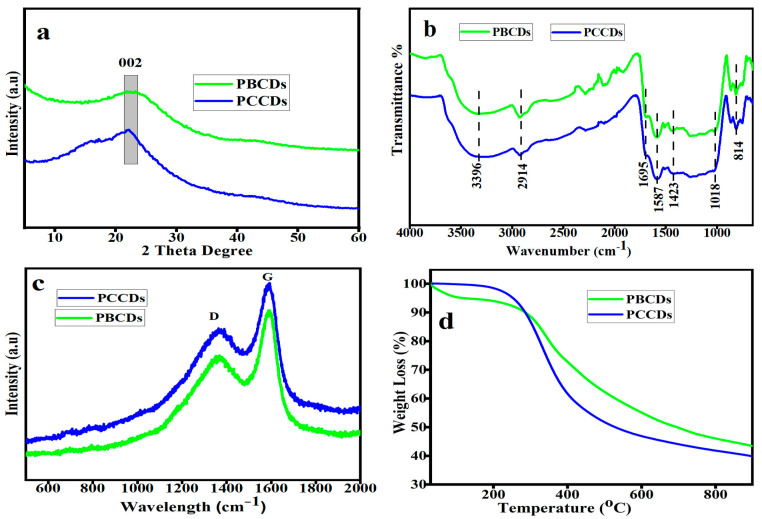
(**a**) XRD patterns, (**b**) FT-IR spectra, (**c**) Raman spectra, and (**d**) TGA of PCCDs and PBCDs.

The XRD pattern (Figure 3a) of the produced PCCDs and PBCDs exhibited amorphous peaks at around 22.1° and 22.6°, which are ascribed to the 002 plane of the graphitic carbon structure [35]. The calculated crystallite sizes for PCCDs and PBCDs were found to be 1.28 and 1.12 nm, respectively. The FTIR spectra of functional groups in the PCCDs and PBCDs (Figure 3b) illustrated a typical broad absorption of the OH stretching band mode at 3396 cm^−1^. The characteristic peak at 2914 cm^−1^ is ascribed to the C-H stretching vibration [36], while the other vibrational stretching peaks of C=O, C=C, and –OCH_3_ are observed at 1695, 1587, and 1423 cm^−1^ [32,37]. Additional stretching peaks of –C-O-C and -CH_2_ appeared at 1018 and 814 cm^−1^. These functionalities, as mentioned earlier, present the PCCDs and PBCDs with excellent fluorescence attributes and water solubility. Figure 3c shows the Raman spectra of the PCCDs and PBCDs, with distinct peaks at 1374 cm^−1^ (D band) and 1585 cm^−1^ (G band), respectively. The D band is assigned to the disordered surface, while the G band corresponds to the graphite carbon of CDs. The ID/IG intensity ratios of PCCDs and PBCDs were calculated to be 0.78 and 0.81, thus confirming the defective graphitic carbon [38]. The thermogravimetric analysis (Figure 3d) of the CDs produced from pine trees, with a minute weight loss (0.24%) for the PCCDs, occurs between 30 and 150 °C. In the case of PBCDs, the weight loss (4.32%) within this range was more pronounced, attributed to surface-adsorbed water molecules’ degradation [39,40]. The second weight loss within the range of 290–600 °C is attributed to the thermal decomposition of oxygenated functional groups bonded to the surface of PCCDs and PBCDs [32]. In this range, the PCCDs have more weight loss (41.55%) than the PBCDs (34.36%).

### 3.2. Optical Performance of the CDs

**Figure 4:**
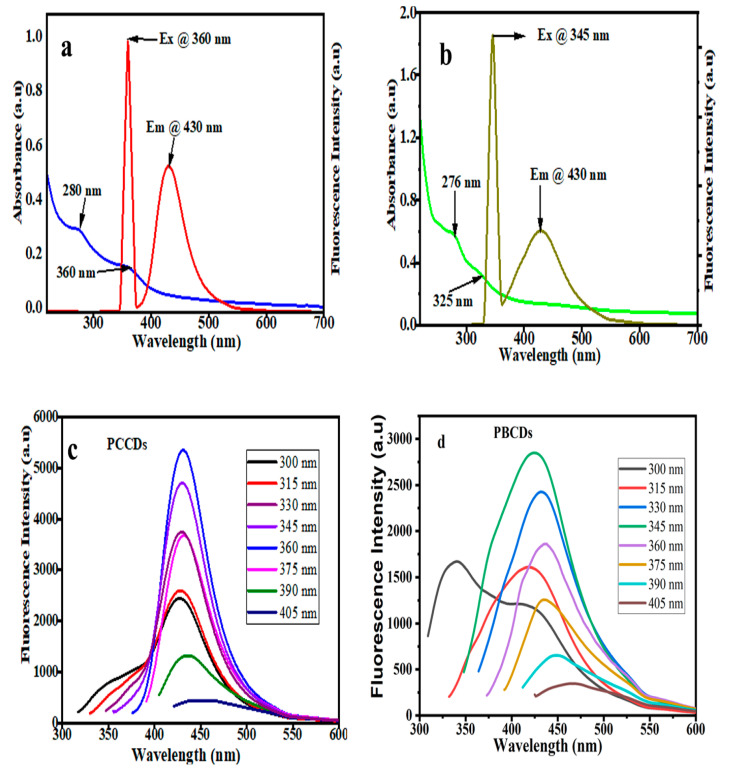
(**a**,**b**) UV−vis spectra and (**c**,**d**) fluorescence excitation and emission spectrum of PCCDs and PBCDs (in ultrapure water solution; detection time: 5 min; and detection temperature: 25 °C).

The optical properties of the synthesized CDs were explored using UV-vis absorption and photoluminescence (PL) spectroscopy. The UV-vis spectra, as presented in Figure 4a,b, evidenced a noticeable absorption band within the 276 to 280 nm range, which is ascribed to the π–π* transition of the aromatic sp^2^ domains [35]. A shoulder absorption peak of around 325–360 nm for both CDs is attributed to n–π* transitions of C=O and the structural defects of the CDs [41]. In addition, the optimal excitation wavelengths of PCCDs and PBCDs are 360 nm and 345 nm, whilst the emission wavelength was pronounced at 430 nm for both CDs. To understand the wavelength-dependent fluorescence properties, the PL properties of the prepared CDs (Figure 4c,d) were varied at different excitation wavelengths within the range of 300 to 405 nm. There was a significant red-shifting in the emission intensity and a decreased fluorescence intensity as the excitation wavelength increased, ascribed to quantum effect and surface defects [42]. This is ascribed to the variation in complex structures present in the biomass precursors, thus resulting in a molecular transition mode change when excited at different wavelengths, subsequently causing the emission wavelength deviation [43]. The quantum yields of synthesized PCCDs and PBCDs by taking Rhodamine B as a reference [32,44] were calculated to be 11.3% and 5.64%.

### 3.3. The Stability Studies

**Figure 5:**
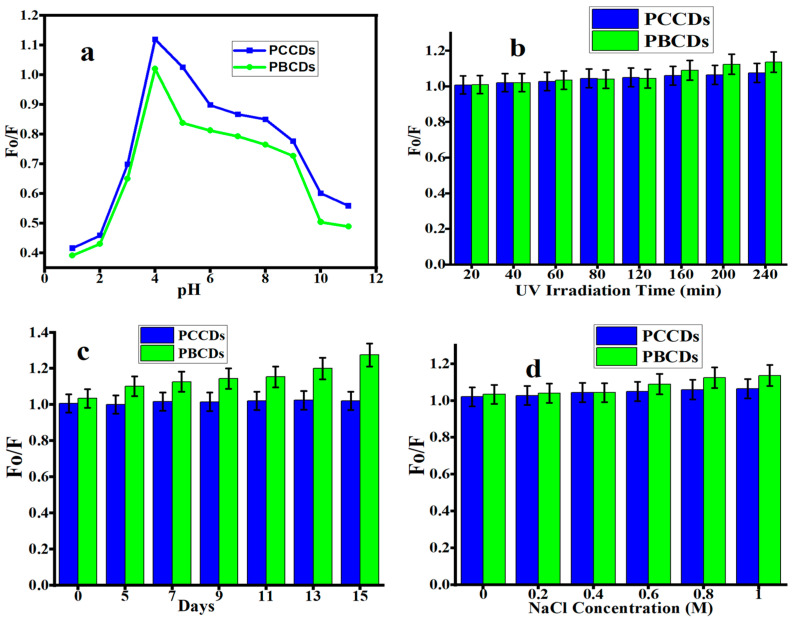
Fluorescence intensity ratios (Fo/F) of PCCDs and PBCDs at different (**a**) pH, (**b**) UV light irradiation, (**c**) storage days, and (**d**) NaCl concentration (detection time: 5 min and detection temperature: 25 °C).

The study of the stability of the PCCDs and PBCDs was carried out under different conditions (under acidic and alkali environments; UV lamp, normal light, and sodium chloride (NaCl) solution) [45,46]. The influence of pH (1 to 11) on the fluorescent emission intensity of PCCDs and PBCDs is presented in Figure 5a. The fluorescence intensity ratios (Fo/F; herein the Fo and F correspond to the fluorescent intensity of prepared CDs in the absence and presence of any additives) of the as-prepared CDs increased when the pH varied from 1 to 4, then started to decline at a higher pH. A pH of 4 yielded the highest fluorescent intensity, and was also found in another work [38,47]. This observation is attributed to different protonation degrees of different functionalities at various pHs [34]. At a pH of below 4 and pH of above 4, the low fluorescence intensity for both PCCDs and PBCDs is caused by the protonation/deprotonation of the surface functional group in different acid and alkaline solutions [32,34]. As shown in Figure 5b,c, both CDs possess exceptional stability with no more than a 10% (PCCDs) and 25% (PBCDs) decline in the original fluorescent intensity under the continuous irradiation of a UV lamp (365 nm) for 4 h and under normal light for 15 days without showing any sign of photobleaching. The PCCDs and PBCDs possessed remarkable stability to adapt to the addition of an NaCl solution with diverse concentrations, as presented in Figure 5d. The sensitivity to pH variation and good photostability properties of PCCDs and PBCDs imply that these materials have great potential for sensing and biological applications under longer exposure durations. The developed method was validated following the guidelines outlined in ICHQ2(R1) to ascertain its dependability and precision [48,49].

### 3.4. Application of PCCDs and PBCDs as Fluorescent Probes

#### 3.4.1. Fluorescence Detection of Tetracycline and Amoxicillin

**Figure 6:**
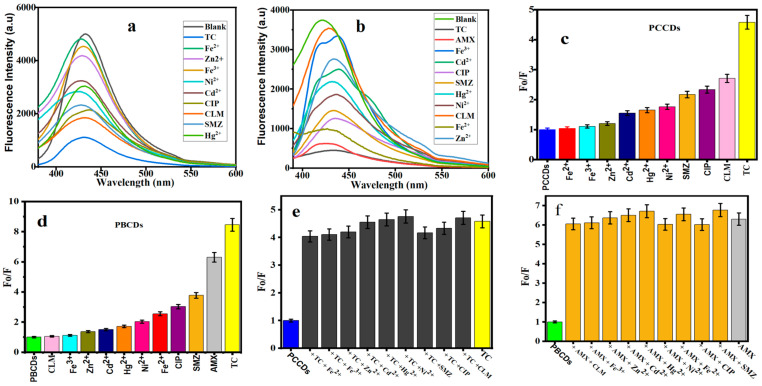
Fluorescence spectra of (**a**) PCCDs and (**b**) PBCDs with different substances. The Fo/F histogram of (**c**) PCCDs and (**d**) PBCDs upon addition of different substrates. Interference studies of (**e**) PCCDs–TC (20 μM) and (**f**) PBCDs–AMX (20 μM) with other substrates (100 μM).

The fluorescent sensors of PCCDs and PBCDs were further utilized to determine their optical properties on some selected metal ions and pharmaceutical antibiotics. Figure 6a,b present the fluorescent emission intensities of PCCDs (@342 nm) and PBCDs (@345 nm). Upon adding these metal ions and pharmaceutical antibiotics, a noticeable diminishing of the intensity is observed (Figure 6a,b). The fluorescent intensity ratios with some antibiotics (CLM, CIP, and SMZ) were slightly reduced between 54 and 64% for the PCCDs, whilst the strongest quenching was evident with TC (78%), as presented in Figure 6c. When other substrates were mixed with TC, the influence of these coexistent interferences was negligible, thus confirming the specificity, selectivity, and sensitivity of PCCDs and PBCDs towards TC detection, as presented in Figure 6e. In the case of PBCDs, Fe^2+^, CIP, and SMZ slightly quenched the emission intensity within the range of 61 to 74%, as presented in Figure 6d. The enhanced quenching is attributed to the nonspecific interactions between the surface functional groups and the antibiotics. However, both TC and AMX impacted the fluorescent intensity ratio for PBCDs, with a more than a 83% reduction (Figure 6d). However, the selectivity of PBCDs towards TC (at different concentrations) in this study was very poor, hence more attention is given to the selective detection of AMX with the PBCD sensor. Also, more focus is projected on the detection of TC and AMX using PCCDs with excellent selectivity. Subsequently, different concentrations of TC (5–100 μM) were added to the PCCDs and PBCDs, and the fluorescent intensity was measured within the range of 340–345 nm. The same approach applies to PBCD selectivity for AMX detection. In addition, interference experiments in the presence of metal ions and other antibiotics were carried out for PBCDs with AMX (20 μM) and other interfering substances (100 μM). The co-existence of other interfering substrates had no obvious influence on the sensitivity or specificity of PBCDs towards AMX, as presented in Figure 6f.

#### 3.4.2. Sensitive Detection of TC by PCCDs and PBCDs

**Figure 7:**
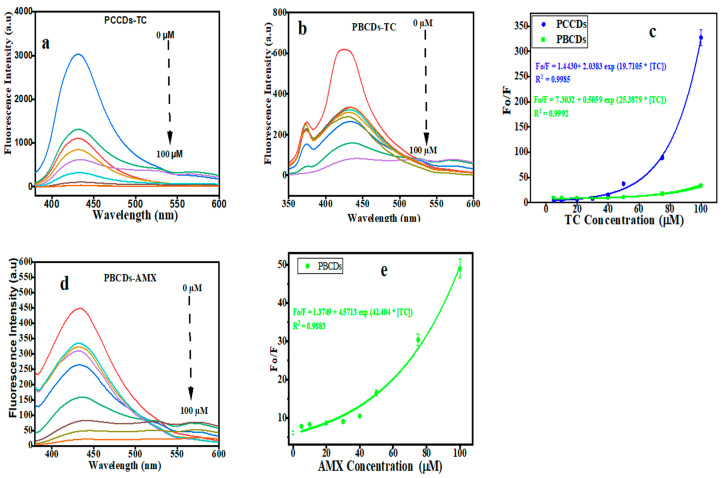
Fluorescence emission intensity of (**a**) PCCDs and (**b**,**d**) PBCDs in the presence of increasing concentrations of tetracycline (TC) and amoxicillin (AMX). Non-linear relationship between Fo/F and the (**c**) TC and (**e**) AMX concentration (5, 10, 20, 30, 40, 50, 75, and 100 μM).

The fluorescence intensity for PCCDs and PBCDs gradually decreases with increased TC concentrations (5, 10, 20, 30, 40, 50, 75, and 100 μM), as presented in Figure 7a,b. It highlights that the PCCD and PCBD systems are very sensitive to the TC concentration. From the plot of the concentrations of TC against Fo/F, it was observed that intensity ratios showed a perfect non-linear relationship with the modified Stern−Volmer expression [46], as displayed in Figure 7c. The non-linear exponential decay was utilized for the curve fitting, like in the previously reported study [32,50]. The experiment data were fitted to the equation Fo/F = 1.4430 + 2.0383 exp 19.7105 * [TC], with a correlation factor (R^2^) of 0.9985 for PCCDs (Figure 7c), while the PBCDs (Figure 7c) present Fo/F = 7.30312 + 0.5059 exp 25.3879 * [TC], with R^2^ value of 0.992. Herein, the Fo and F represent the fluorescent intensity of either the PCCD or PBCD solution in the absence or presence of TC or AMX at different concentrations. The limit of detection (LOD, at the signal-to-noise ratio of 3: S/N = 3) for the PCCDs and PBCDs, as calculated from the non-linear curve, was estimated to be 0.062 μM and 0.2237 μM, respectively. The PCCDs present a better LOD in comparison with PBCDs in the sensitive detection of TC, which is largely attributed to the quantum yield. The detection limit for TC with the PCCD and PBCD probes is comparable with or better than in the previously reported studies in the literature (Table 1).

#### 3.4.3. Sensitive Detection of AMX by PBCDs

The fluorescence emission spectra for PBCDs were recorded, while different concentrations of AMX were added to the PBCD solution. The fluorescence intensity for PBCDs has shown a gradual decline, owing to the sensitive detection of the AMX probe (Figure 7d). In the range of 5 to 100 μM, the PCBDs showed a perfect non-linear relationship with the fitted equations, as Fo/F = 1.3749 + 4.5713 exp 42.4104 * [AMX], with a correlation factor (R^2^) of 0.9883 (Figure 7e). The LOD of PBCDs to AMX (S/N = 3) was as low as 0.4909 μM. We also compared the results with previous studies and concluded that the PBCDs had a better LOD at a wider range in AMX detection (Table 2).

### 3.5. Detection Mechanism

**Figure 8:**
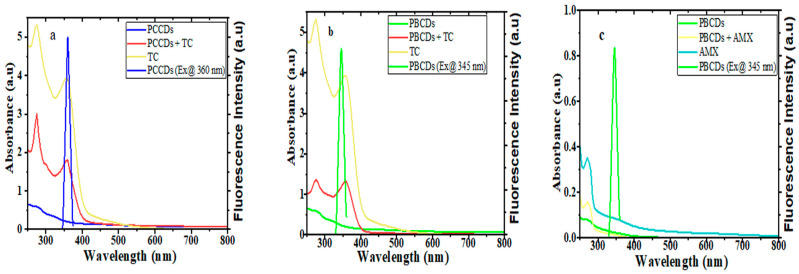
UV-visible absorption spectra and fluorescence emission spectra of (**a**) PCCDs-TC, (**b**) PBCDs-TC, and (**c**) PBCDs–AMX (detection time: 5 min and detection temperature: 25 °C).

Further inquiries regarding the quenching mechanism of the PCCDs and PBCDs in tandem with TC and AMX were executed using the UV absorption and fluorescence studies. The absorption peak intensities of PCCDs and PBCDs were enhanced upon the addition of TC, without any deviation in the wavelength, thus implying no formation of new substances (Figure 8a,b). Furthermore, the UV spectra of TC showed that the absorption behavior overlapped with the excitation wavelengths of PCCDs and PBCDs in our studies at around 360 nm (Figure 8a,b). On the premise that the UV spectra of TC overlapped with the excitation wavelength of the prepared CDs, the existence of an internal filter effect (IFE) [60] between TC and synthesized CDs from pine tree was proven. Furthermore, we had a slightly enhanced absorption spectrum of PBCDs when AMX was introduced into the system (Figure 7c). However, there was a huge disparity with the absorption spectra of PBCDs–AMX, not overlapping with the excitation wavelength of PBCDs (Figure 8c), and thus indicating that the IFE was not applicable here. Therefore, the quenching of PBCDs by AMX is preliminarily inferred to be due to a static quenching process.

### 3.6. Real Water Analysis

The recovery studies were executed to evaluate the practicality and reliability of the sensing method in real water samples obtained from tap water within the “Vaal University of Technology” campus (Vanderbijlpark). Herein, different concentrations (15, 20, and 25 μM) of TC and AMX were spiked into the tap water in the presence of prepared CDs, and the results are presented in Table 3. No TC or AMX was detected in the tap water sample alone, suggesting that the tap water did not initially contain these antibiotics and had no intrusion on the CD response. Table 3 shows the satisfactory recoveries for detecting TC and AMX, ranging from 96.12% to 102.74%, with relative standard deviations (RSDs) of less than 2%. The recoveries and RSD are determined to be 96.12–102.74% and less than 2%, respectively.

## 4. Conclusions

In this work, we have utilized pinecones and pine bark as waste biomass in the preparation of pinecone carbon dots (PCCDs) and pine bark carbon dots (PBCDs) through the microwave pyrolysis route. Both CDs evidence many surface functional groups, reinforced by the FTIR and thermal analysis. In addition, the CDs presented excitation wavelength dependence, good stability, and quantum yields within the range of 6 to 11%. PCCDs and PBCDs showed “turn-off” detection of TC and AMX by quenching the fluorescent intensity. The limits of detection (LOD) for TC at a wider concentration range, using PCCDs and PBCDs, were 0.062 and 0.2237 µM, respectively. PBCDs utilized as fluorescent probes for selective AMX detection presented an LOD of 0.49 µM, and the detection obeyed the static quenching mechanism. Furthermore, the prepared CDs (PCCDs and PBCDs) were successfully applied in the detection of TC and AMX antibiotics in actual water samples.

## Figures and Tables

**Table 1:** Comparison of different fluorescent probes for TC detection.

Probes	Precursors	Linear Range (μM)	Detection Limit (μM)	Reference
CDs	o-phenylenediamine	10.0–400.0	6.0	[51]
MIPs-AA/CQDs	Citric acid Polyethyleneimine	1–60	0.17	[52]
CDs	Citric acid Ethylenediamine	1–300	0.30	[53]
GUCDs	Glycerol Urea	0.5–25	0.165	[54]
RBP-CDs	Red beet pigment	0.5–90	0.36	[40]
CRSSs-NH_2_@N-CDs	Aminated central radial silica spheres	0.5–60	0.39	[55]
PCCDs, PBCDs	Pinecones and Pine Bark	5–100	0.062 and 0.2237	This work

**Table 2:** Different fluorescent probes reported for the detection of AMX.

Probes	Precursors	Linear Range (μM)	Detection Limit (μM)	Reference
N, S-CDs-Sop	S. japonica flower	5–80	3.40	[56]
CDs@CdTe@MIP			0.15	[57]
Cu, N@CQDs		0.2–120.0	0.06	[58]
B-CQDs	Citric acid monohydrate and boric acid	1.43–429.12	0.825	[59]
PBCDs	Pine Bark	5–100	0.49	This work

**Table 3:** Determination of TC and AMX in real water samples.

Water Sample	Added (µM)	Found (µM)	Recovery (%)	RSD (%, n = 3)
	0	ND		
	15	15.4106	102.7374	1.1059
Tap Water (TC)	20	19.2239	96.1199	1.1211
	25	25.4194	101.6776	1.1362
	0	ND		
	15	14.8078	98.7192	0.3549
Tap Water (AMX)	20	20.1679	100.8396	0.3617
	25	24.7673	99.0691	0.3652

ND: not detected.

## Data Availability

The original contributions presented in this study are included in the article. Further inquiries can be directed to the corresponding author(s).

## References

[1] Priya B., Shandilya P., Raizada P., Thakur P., Singh N., Singh P. (2016). Photocatalytic mineralization and degradation kinetics of ampicillin and oxytetracycline antibiotics using graphene sand composite and chitosan supported BiOCl. J. Mol. Catal. A Chem..

[2] Raizada P., Kumari J., Shandilya P., Singh P. (2017). Kinetics of photocatalytic mineralization of oxytetracycline and ampicillin using activated carbon supported ZnO/ZnWO_4_ nanocomposite in simulated wastewater. Desalination Water Treat..

[3] Adil M., Iqbal M., Kanwal S., Abbas G. (2024). Toxicity and Adverse Effects of Veterinary Pharmaceuticals in Animals. Pharmaceuticals in Aquatic Environments.

[4] Bayode A.A., Osti A., Glisenti A. (2024). Sonophotocatalytic degradation of sulfamethoxazole using lanthanum ferrite perovskite oxide anchored on an ultrasonically exfoliated porous graphitic carbon nitride nanosheet. RSC Adv..

[5] Sodhi K.K., Kumar M., Singh D.K. (2021). Insight into the amoxicillin resistance, ecotoxicity, and remediation strategies. J. Water Process Eng..

[6] Ayanda O.S., Mmuoegbulam A.O., Okezie O., Durumin Iya N.I., Mohammed S.a.E., James P.H., Muhammad A.B., Unimke A.A., Alim S.A., Yahaya S.M. (2024). Recent progress in carbon-based nanomaterials: Critical review. J. Nanoparticle Res..

[7] Fritea L., Banica F., Costea T.O., Moldovan L., Dobjanschi L., Muresan M., Cavalu S. (2021). Metal nanoparticles and carbon-based nanomaterials for improved performances of electrochemical (Bio) sensors with biomedical applications. Materials.

[8] Ozyurt D., Al Kobaisi M., Hocking R.K., Fox B. (2023). Properties, synthesis, and applications of carbon dots: A review. Carbon Trends.

[9] Khan M.E., Mohammad A., Yoon T. (2022). State-of-the-art developments in carbon quantum dots (CQDs): Photo-catalysis, bio-imaging, and bio-sensing applications. Chemosphere.

[10] Raeispour S., Rahmandoust M., Kouchakzadeh H. (2024). A nanocarrier system based on CQDs for efficient mitoxantrone drug delivery. Heliyon.

[11] Yadav P.K., Chandra S., Kumar V., Kumar D., Hasan S.H. (2023). Carbon quantum dots: Synthesis, structure, properties, and catalytic applications for organic synthesis. Catalysts.

[12] Manikandan V., Lee N.Y. (2022). Green synthesis of carbon quantum dots and their environmental applications. Environ. Res..

[13] Islam R., Ahammad R., Islam M.M., Shoeb M., Mamun M.I.R. (2024). High performance liquid chromatography assessment of antibiotic residues in poultry and fish feeds in Bangladesh. Curr. Res. Biosci. Biotechnol..

[14] Montone C.M., Moneta B.G., Laganà A., Piovesana S., Taglioni E., Cavaliere C. (2024). Transformation products of antibacterial drugs in environmental water: Identification approaches based on liquid chromatography-high resolution mass spectrometry. J. Pharm. Biomed. Anal..

[15] Liu B., Zhu H., Feng R., Wang M., Hu P., Pan J., Niu X. (2022). Facile molecular imprinting on magnetic nanozyme surface for highly selective colorimetric detection of tetracycline. Sens. Actuators B Chem..

[16] Wang X., Xie Y., Lin L. (2022). Recent development of microfluidic biosensors for the analysis of antibiotic residues. TrAC Trends Anal. Chem..

[17] Ramzan M., Raza A., un Nisa Z., Abdel-Massih R.M., Al Bakain R., Cabrerizo F.M., Cruz T.E.D., Aziz R.K., Musharraf S.G. (2024). Detection of antimicrobial resistance (AMR) and antimicrobial susceptibility testing (AST) using advanced spectroscopic techniques: A review. TrAC Trends Anal. Chem..

[18] El-Ghobashy M.R., Abo-Talib N.F. (2010). Spectrophotometric methods for the simultaneous determination of binary mixture of metronidazole and diloxanide furoate without prior separation. J. Adv. Res..

[19] Imran M., Ahmed S., Abdullah A.Z., Hakami J., Chaudhary A.A., Rudayni H.A., Khan S.U.D., Khan A., Basher N.S. (2023). Nanostructured material-based optical and electrochemical detection of amoxicillin antibiotic. Luminescence.

[20] Zhao Y., Geng X., Shi X., Guo Y., Sun Y., Qu L., Li Z. (2021). A fluorescence-switchable carbon dot for the reversible turn-on sensing of molecular oxygen. J. Mater. Chem. C.

[21] Amjadi M., Jalili R. (2018). A molecularly imprinted dual-emission carbon dot-quantum dot mesoporous hybrid for ratiometric determination of anti-inflammatory drug celecoxib. Spectrochim. Acta Part. A Mol. Biomol. Spectrosc..

[22] Li S., Li L., Tu H., Zhang H., Silvester D.S., Banks C.E., Zou G., Hou H., Ji X. (2021). The development of carbon dots: From the perspective of materials chemistry. Mater. Today.

[23] Xia C., Zhu S., Feng T., Yang M., Yang B. (2019). Evolution and synthesis of carbon dots: From carbon dots to carbonized polymer dots. Adv. Sci..

[24] Hui S. (2023). Carbon dots (CDs): Basics, recent potential biomedical applications, challenges, and future perspectives. J. Nanoparticle Res..

[25] Othman K.A., Ali L.I.A., Qader A.F., Omer R.A., Amin A.A. (2024). Synthesis, Characterization, and Applications of Carbon Dots for Determination of Pharmacological and Biological Samples: A Review. J. Fluoresc..

[26] Antar M., Lyu D., Nazari M., Shah A., Zhou X., Smith D.L. (2021). Biomass for a sustainable bioeconomy: An overview of world biomass production and utilization. Renew. Sustain. Energy Rev..

[27] Ali F., Dawood A., Hussain A., Alnasir M.H., Khan M.A., Butt T.M., Janjua N.K., Hamid A. (2024). Fueling the future: Biomass applications for green and sustainable energy. Discov. Sustain..

[28] Gan J., Chen L., Chen Z., Zhang J., Yu W., Huang C., Wu Y., Zhang K. (2023). Lignocellulosic biomass-based carbon dots: Synthesis processes, properties, and applications. Small.

[29] Wangmo D., Rana R. (2024). Pine Needles as Green Material for Removal of Metal Ions and Dyes from Wastewater. Green Chemistry in Environmental Sustainability and Chemical Education.

[30] Liu Y., Ge G., Liu H., Wang Y., Zhou P., Li B., Zhu G. (2024). Fast and eco-friendly synthesis of carbon dots from pinecone for highly effective detection of 2, 4, 6-trinitrophenol in environmental samples. Environ. Technol..

[31] Shahba H., Sabet M. (2020). Two-step and green synthesis of highly fluorescent carbon quantum dots and carbon nanofibers from pine fruit. J. Fluoresc..

[32] Sanni S.O., Moundzounga T.H.G., Oseghe E.O., Haneklaus N.H., Viljoen E.L., Brink H.G. (2022). One-Step Green Synthesis of Water-Soluble Fluorescent Carbon Dots and Its Application in the Detection of Cu^2+^. Nanomaterials.

[33] Miller J., Miller J.C. (2018). Statistics and Chemometrics for Analytical Chemistry.

[34] Das P., Maruthapandi M., Saravanan A., Natan M., Jacobi G., Banin E., Gedanken A. (2020). Carbon Dots for Heavy-Metal Sensing, pH-Sensitive Cargo Delivery, and Antibacterial Applications. ACS Appl. Nano Mater..

[35] Wang Y., Liu Y., Zhao L., Sun L., Zhao X., Xia Y. (2021). κ-Carrageenan-derived carbon dots for highly selective and sensitive detection of Fe^3+^ and oxytetracycline. J. Mater. Sci..

[36] Chaudhary S., Kumari M., Chauhan P., Ram Chaudhary G. (2021). Upcycling of plastic waste into fluorescent carbon dots: An environmentally viable transformation to biocompatible C-dots with potential prospective in analytical applications. Waste Manag..

[37] Krishnaiah P., Atchudan R., Perumal S., Salama E.-S., Lee Y.R., Jeon B.-H. (2022). Utilization of waste biomass of Poa pratensis for green synthesis of n-doped carbon dots and its application in detection of Mn^2+^ and Fe^3+^. Chemosphere.

[38] Wang J., Fu J., Chen M., Zhang J. (2024). Red-emitting carbon dots for fluorescent and smartphone-assisted dual-mode detection of Cu(II), Hg(II), and Fe(III) and investigation of the sensing mechanism. Mater. Today Chem..

[39] Sanni S.O., Viljoen E.L., Ofomaja A.E. (2020). Three-dimensional hierarchical porous carbon structure derived from pinecone as a potential catalyst support in catalytic remediation of antibiotics. RSC Adv..

[40] Cao Y., Wang X., Bai H., Jia P., Zhao Y., Liu Y., Wang L., Zhuang Y., Yue T. (2022). Fluorescent detection of tetracycline in foods based on carbon dots derived from natural red beet pigment. LWT.

[41] Jessy Mercy D., Kiran V., Thirumalai A., Harini K., Girigoswami K., Girigoswami A. (2023). Rice husk assisted carbon quantum dots synthesis for amoxicillin sensing. Results Chem..

[42] Sun P., Song W., Zou Y., Tian M., Zhang F., Chai F. (2023). The fabrication of N-doped carbon dots by methionine and their utility in sensing Cu^2+^ in real water. Anal. Methods.

[43] Qi H., Liu C., Jing J., Jing T., Zhang X., Li J., Luo C., Qiu L., Li Q. (2022). Two kinds of biomass-derived carbon dots with one-step synthesis for Fe^3+^ and tetracyclines detection. Dye. Pigment..

[44] Li C., Liu L., Zhang D. (2024). Aggregation enhanced emissive orange carbon dots for information encryption and detection of Fe^3+^ and tetracycline. Spectrochim. Acta Part A Mol. Biomol. Spectrosc..

[45] Wang K., Ji Q., Xu J., Li H., Zhang D., Liu X., Wu Y., Fan H. (2018). Highly sensitive and selective detection of amoxicillin using carbon quantum dots derived from beet. J. Fluoresc..

[46] Kaur N., Tiwari P., Abbas Z., Mobin S.M. (2022). Doxycycline detection and degradation in aqueous media via simultaneous synthesis of Fe-N@ carbon dots and Fe_3_O_4_-carbon dot hybrid nanoparticles: A one arrow two hawk approach. J. Mater. Chem. B.

[47] Mathew S., John B.K., Mathew J., Korah B.K., Mathew D.B. (2023). Green synthesized carbon dots as antibiotics sensor and fluorescent ink. J. Mol. Struct..

[48] Borman P., Elder D. (2017). Q2 (R1) validation of analytical procedures: Text and methodology. ICH Quality Guidelines: An Implementation Guide.

[49] Somatek (2005). Validation of analytical procedures: Text and methodology. Q2 (R1). ICH Harmonised Tripartite Guideline.

[50] Mu Y., Zhuang Q., Huang S., Hu M., Wang Y., Ni Y. (2020). Adenine-stabilized carbon dots for highly sensitive and selective sensing of copper (II) ions and cell imaging. Spectrochim. Acta Part A Mol. Biomol. Spectrosc..

[51] Yan Y., Liu J.H., Li R.S., Li Y.F., Huang C.Z., Zhen S.J. (2019). Carbon dots synthesized at room temperature for detection of tetracycline hydrochloride. Anal. Chim. Acta.

[52] Wei X., Lv L., Zhang Z., Guan W. (2020). Preparation of molecularly imprinted fluorescence sensor based on carbon quantum dots via precipitation polymerization for fluorescence detection of tetracycline. J. Appl. Polym. Sci..

[53] Long D., Peng J., Peng H., Xian H., Li S., Wang X., Chen J., Zhang Z., Ni R. (2019). A quadruple-channel fluorescent sensor array based on label-free carbon dots for sensitive detection of tetracyclines. Analyst.

[54] Uriarte D., Domini C., Garrido M. (2019). New carbon dots based on glycerol and urea and its application in the determination of tetracycline in urine samples. Talanta.

[55] Yuan M., An J., Zhang G., Hu Y., Luo M., Shi Y., Liu Y. (2022). In-situ nitrogen-doped carbon dots for fluorescence sensing of tetracycline antibiotic. Ceram. Int..

[56] Guo X.-R., Dong Y.-M., Chen X.-Y., Chen J. (2022). *Sophorajaponica* L. flower mediated carbon dots with nitrogen and sulfur co-doped as a sensitive fluorescent probe for amoxicillin detection. Spectrochim. Acta Part A Mol. Biomol. Spectrosc..

[57] Li C., Ma Y., Fan C., He C., Ma S. (2024). Highly sensitive and selective detection of amoxicillin using molecularly imprinted ratiometric fluorescent nanosensor based on quantum dots. Microchim. Acta.

[58] Mahmoud A.M., El-Wekil M.M., Ali R., Batakoushy H.A., Shahin R.Y. (2022). Double-signal quantification of amoxicillin based on interaction with 4-aminoantipyrine at copper and nitrogen co-doped carbon quantum dots as an artificial nanozyme. Microchim. Acta.

[59] Zhang X., Ren Y., Ji Z., Fan J. (2020). Sensitive detection of amoxicillin in aqueous solution with novel fluorescent probes containing boron-doped carbon quantum dots. J. Mol. Liq..

[60] Jia P., Bu T., Sun X., Liu Y., Liu J., Wang Q., Shui Y., Guo S., Wang L. (2019). A sensitive and selective approach for detection of tetracyclines using fluorescent molybdenum disulfide nanoplates. Food Chem..

